# Bilateral Subdural Hygromas After Deep Brain Stimulation Implantation in the Setting of Unrecognized Intracranial Hypotension

**DOI:** 10.7759/cureus.13018

**Published:** 2021-01-30

**Authors:** Juliana Rotter, John Atkinson, Jeremy K Cutsforth-Gregory, Bryan T Klassen, Kai Miller

**Affiliations:** 1 Neurologic Surgery, Mayo Clinic, Rochester, USA; 2 Neurology, Mayo Clinic, Rochester, USA

**Keywords:** cerebrospinal fluid venous fistula, csf venous fistula, dbs, deep brain stimulation, hygroma, csf leak

## Abstract

Cerebrospinal fluid (CSF)-venous fistulas are a recently recognized cause of spontaneous spinal CSF leak and present most commonly with Valsalva (“cough”)-exacerbated or orthostatic headaches. By inducing CSF hypotension, they cause diffuse pachymeningeal enhancement and brain sag on MRI. This unusual case demonstrates the potential for bilateral subdural hygroma development in a patient with an undiagnosed CSF-venous fistula after ventral intermediate nucleus (VIM) deep brain stimulation (DBS) implantation. A 68-year-old gentleman with medically-refractory essential tremor underwent extensive preoperative evaluation by the Mayo Clinic-Rochester DBS Committee. Initial MRI during preoperative evaluation had no evidence of CSF hypotension, but MRI performed the day before surgery demonstrated diffuse pachymeningeal enhancement. He underwent bilateral VIM DBS implantation and presented in the subacute postoperative period with bilateral subdural hygromas. Further testing identified a prominent hyperdense paraspinal vein arising from the T10/T11 nerve root, consistent with CSF-venous fistula. Even when patients undergo rigorous preoperative evaluations for surgical procedures, insidious pathologies can develop and cause unexpected postoperative complications.

## Introduction

Cerebrospinal fluid (CSF)-venous fistulas can cause intracranial hypotension, which radiographically manifests as diffuse pachymeningeal enhancement and brain sagging. Diagnosis of CSF-venous fistulas requires CT or digital subtraction myelography as they are not evident on routine MRI or CT scans [1,2]. These uncommon causes of spontaneous CSF leak often are associated with Valsalva (“cough”)-exacerbated or orthostatic headaches [3]. This unusual case demonstrates the potential for bilateral subdural hygroma development in a patient with an undiagnosed CSF-venous fistula after ventral intermediate nucleus (VIM) deep brain stimulation (DBS) implantation.

## Case presentation

Methods

A retrospective chart review was conducted to describe in detail the clinical history and other pertinent details of the reported case. In tandem, we carried out a review of the literature. Three electronic searches were performed using Ovid Embase, PubMed, SCOPUS, and Cochrane databases from each individual database's inception to June 2020. The first literature search used the following string of terms: (CSF venous fistula) OR (Cerebrospinal Fluid venous fistula) AND (DBS) OR (Deep Brain Stimulation). The second literature search used the following string of terms: (CSF venous fistula) OR (Cerebrospinal fluid venous fistula) AND (hygroma). The third literature search used the following string of terms: (DBS) OR (Deep Brain Stimulation) AND (hygroma). These three literature searches did not identify similar published cases describing postoperative DBS patients who developed bilateral subdural hygromas, which led to the diagnosis of a CSF-venous fistula.

 

Results

A 68-year-old right-handed gentleman with longstanding, medically-refractory essential tremor (ET), with right upper extremity tremor greater than left, presented for consideration of operative management. His symptoms began in high school and progressed despite maximal pharmaceutical management. He had no history of spine surgery, spinal anesthesia, or lumbar puncture. Neuropsychometric, speech, and psychologic evaluations were normal, without contraindications to surgery. Multichannel surface EMG confirmed an organic asymmetric postural and action tremor at 4.5 Hz. On physical exam, his only positive neurologic findings were bilateral tremor, right worse than left, and bilateral worsening of tremor amplitude with intention and action. As part of his work-up, an MRI brain with gadolinium contrast was obtained, which was unremarkable. His case was discussed at the multidisciplinary DBS Committee meeting for consideration of high-frequency ultrasound versus bilateral VIM DBS. Ultimately, DBS was recommended because of superior system adjustability for tremor treatment over a longer period of time and to treat bilateral symptoms.

**Figure 1:**
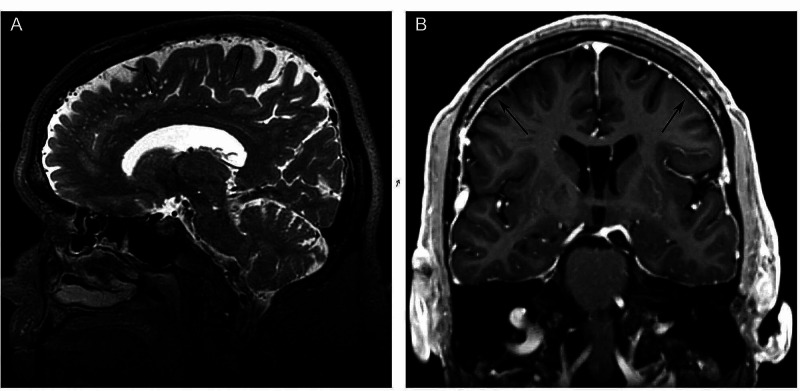
Preoperative MRI. Sagittal T2 (A) and coronal (B) T1 MRI brain with gadolinium contrast demonstrating preoperative mild, diffuse, smooth pachymeningeal enhancement without brain sag.

Six months after the initial MRI, he underwent a repeat preoperative MRI brain with gadolinium contrast the day before surgery, which was co-registered with a non-contrast head CT scan obtained the morning of surgery (Figure 1). During the approach for the bilateral VIM DBS implantation, his arachnoid membrane was noted to be unexpectedly thick, requiring bipolar cautery and microscissors to penetrate. An Alpha Omega micro drive positioned microelectrodes for intraoperative recording of multiple motion-turned neurons and tremor cells, but no sensory-tuned neurons. After the microelectrode was removed, the DBS lead was advanced to target depth and a test simulation demonstrated expected tremor reduction. He tolerated the procedure well and returned on postoperative day 5 for uneventful right-sided infraclavicular neurostimulator placement. 

**Figure 2:**
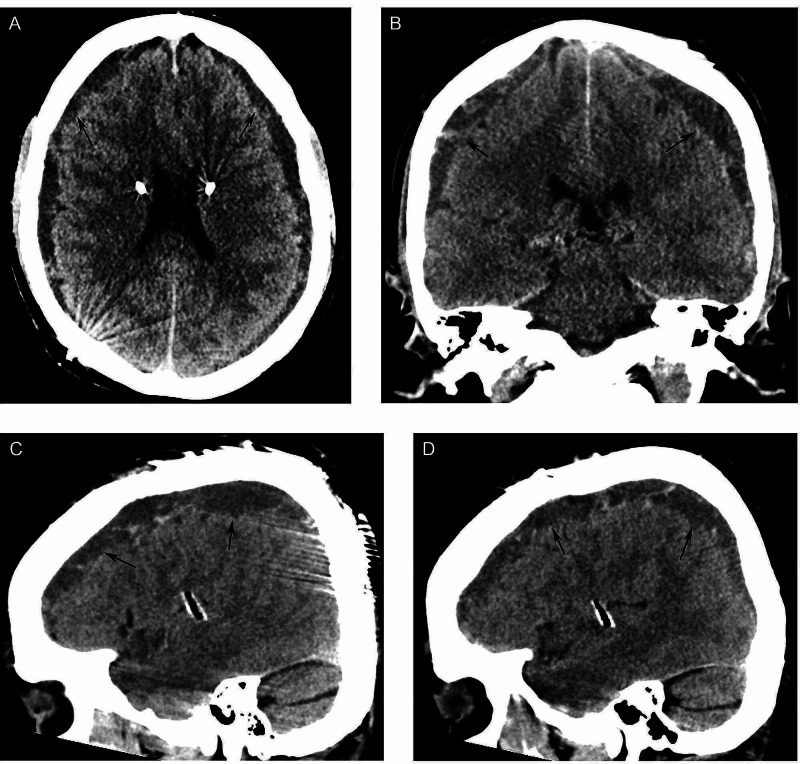
Postoperative head CT. (A) Axial, (B) coronal, (C) right para-sagittal, and (D) left para-sagittal non-contrast head CT from postoperative day 15, showing bilateral hygromas with crescentic area of hyperattenuation in the left parieto-occipital region concerning for acute to subacute subdural hematoma superimposed on layering blood products.

On postoperative day 10, he presented to an outside emergency department for word-finding difficulty and right monocular vision loss, which self-resolved in less than one hour. A non-contrast head CT identified bilateral subdural hygromas (Figure 2). In retrospect, the patient acknowledged a several-month history of headaches from violent coughing episodes as well as mild positional right-sided aching headaches most days, improved by alternating acetaminophen and ibuprofen as needed; the patient did not report aspirin use. Closer review of preoperative imaging identified diffuse pachymeningeal enhancement, a finding associated with intracranial hypotension (Figure 1) [1]. It was at this point that the intraoperative finding of thickened arachnoid membrane was theorized to be the biological response to intracranial hypotension and possible underlying spinal CSF leak.

**Figure 3:**
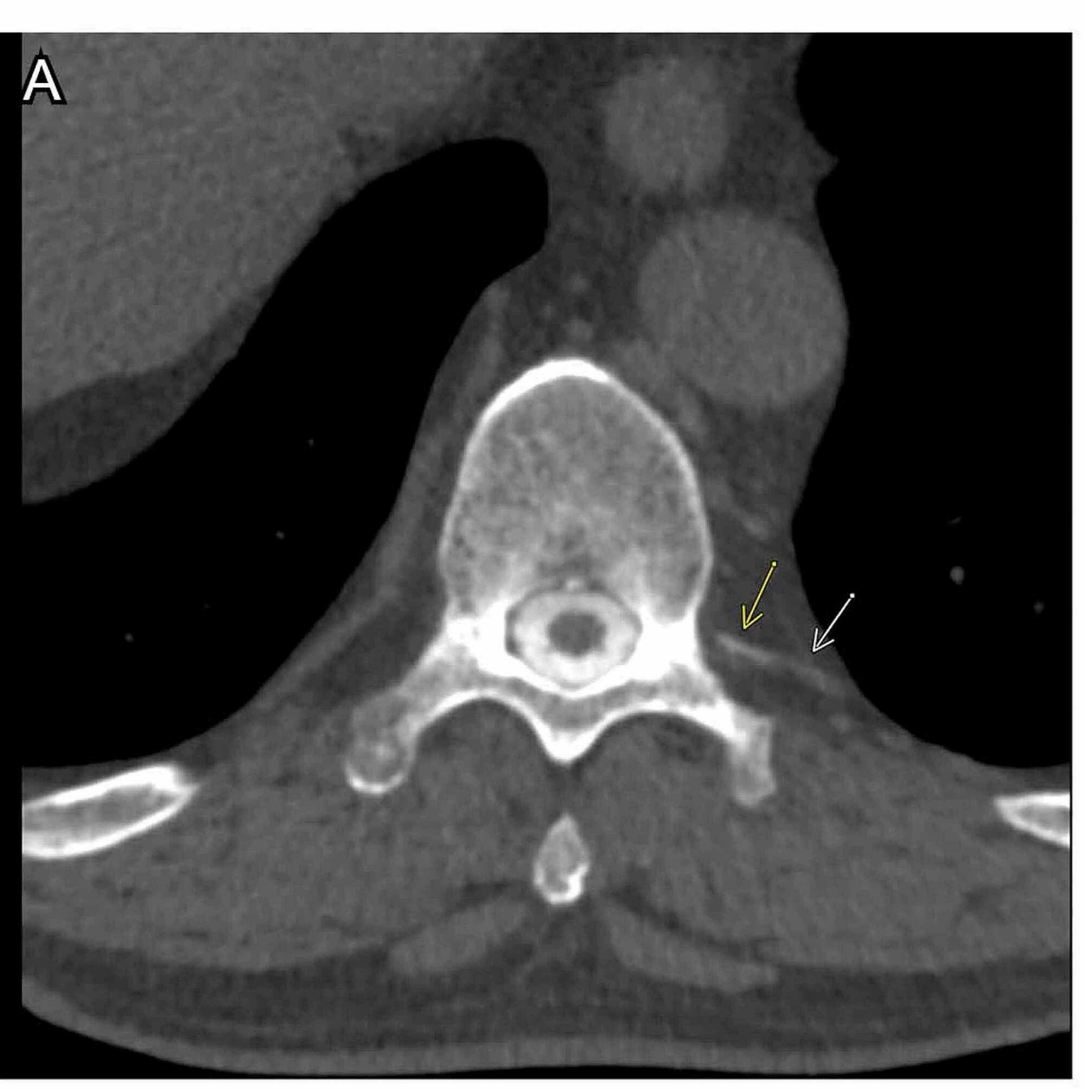
CT myelogram. Axial CT thoracic myelogram showing hyperdense paraspinal vein consistent with CSF-venous fistula arising from left T10-T11 nerve root. CSF: cerebrospinal fluid.

Although operative management of the bilateral hygromas was considered, surgical treatment was deferred until the underlying etiology causing CSF hypotension could be made. The patient remained clinically stable, and the hygromas remained stable on repeat CT scan 6 weeks post-operatively. We felt that, even if evacuated, the collections would likely re-develop if the underlying CSF leak was not corrected. Colleagues in our CSF Dynamics Clinic arranged CT myelogram, which revealed a prominent hyperdense paraspinal vein emanating from the left T10/T11 nerve root sleeve, consistent with a CSF-venous fistula (Figure 3).

**Figure 4:**
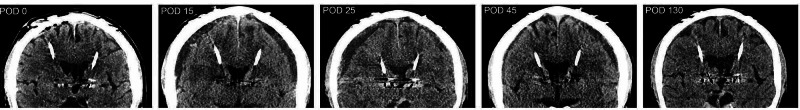
Follow-up non-contrast head CT scans. Coronal slices of non-contrast CT scans performed immediately postoperatively, and at postoperative days 0, 15, 25, 45, and 130 (with slightly different gantries on each scan).

We considered surgical nerve root ligation to treat the CSF-venous fistula. The COVID-19 pandemic delayed elective surgery. During the forced period of observation, he remained clinically stable without further neurological episodes, and the hygromas resolved spontaneously over four months (Figure 4). At the time of publication, he appreciated more than an 80% tremor reduction with DBS.

## Discussion

Serious surgical complications from DBS, including intracranial hemorrhage or ischemic events, are rare, with 0.4% 30-day perioperative mortality and 1% permanent neurologic morbidity [4]. The most common adverse events in patients with ET include dysarthria, ataxia, pain, paresthesia, asthenia, insomnia, hypophonia, somnolence, and dysphagia; postoperative unilateral subdural hematomas are very rare [5]. One case of transient stimulation-associated central nystagmus has been reported after VIM DBS for ET [6]. Our case of bilateral subdural hygromas developing after VIM DBS for ET is unique. Since the pachymeningeal enhancement arose during the six months between preoperative imaging and surgery, it seems likely that the patient developed a CSF-venous fistula in the setting of vigorous coughing fits. By causing CSF hypotension, the fistula increased the risk of postoperative subdural hygromas. Since the development of unilateral subdural hematomas after DBS implantation is unusual, this patient's bilateral subdural hygromas more likely resulted from fistula-induced intracranial hypotension. In most patients, the underlying cause of CSF hypotension is a spinal CSF leak [7]. Once in the epidural space, CSF is absorbed by the spinal epidural venous plexus, which are maximally dilated in the setting of CSF hypotension and can often be seen on conventional MRI of the spine [8]. Treatment options include sacrificing a nonappendicular nerve root or venous communication obliteration when affected nerve roots cannot be sacrificed without causing unacceptable neurologic deficits [1].

## Conclusions

Between the completion of the thorough preoperative evaluation for surgical management of our patient’s ET and his bilateral VIM DBS implantation, this patient likely developed a spontaneous CSF venous fistula that led to CSF hypotension and predisposed him to postoperative bilateral subdural hygromas though bilateral postoperative subdural hematomas cannot be completely ruled out. An increased duration of time between initial evaluation and surgical management adds risk of new pathologies developing that may contribute to unexpected postoperative complications. Furthermore, the increased number of indications for and the greater frequency of DBS surgeries may result in increased identification of rare and unusual complications that require prompt diagnosis and disease-specific treatment.
